# Endoplasmic reticulum stress at the crossroads of progeria and atherosclerosis

**DOI:** 10.15252/emmm.201910360

**Published:** 2019-03-22

**Authors:** Elisa Di Pasquale, Gianluigi Condorelli

**Affiliations:** ^1^ Institute of Genetic and Biomedical Research (IRGB) – Milan Unit National Research Council Milan Italy; ^2^ Humanitas Clinical and Research Center – IRCCS Rozzano Milan Italy; ^3^ Humanitas University Milan Italy

**Keywords:** Ageing, Genetics, Gene Therapy & Genetic Disease, Vascular Biology & Angiogenesis

## Abstract

Hutchinson–Gilford progeria syndrome (HGPS) is a rare pathology caused by a specific mutation (*c*.1824C>T; *p*.G608G) in the *LMNA* gene (Eriksson *et al*, 2003). In healthy conditions, *LMNA* encodes lamins A and C, two major structural nuclear proteins. The mutation creates a splice site in exon 11, resulting in ubiquitous expression of progerin, an aberrant lamin A precursor. Mutations of *LMNA* can cause laminopathies, a group of diseases with a wide spectrum of, often overlapping, tissue‐specific phenotypes. HGPS is probably one of the most devastating forms of laminopathy. Affected patients display signs of accelerated aging, such as lack of subcutaneous fat, hair loss, joint contractures, and skin thinning, and usually die prematurely from cardiovascular complications. Atherosclerosis is one of the most severe and clinically relevant features of HGPS, manifesting in the absence of classical risk factors, such as increased low‐density lipoprotein and C‐reactive protein (Gordon *et al*, 2005). In this issue, Hamczyk *et al* (2019) describe a mechanism for HGPS‐related atherosclerosis.

It has been extensively demonstrated that accumulation of progerin is extremely toxic for cells, causing progressive molecular defects. The proposed mechanisms have been based on mouse models and various primary and reprogrammed cells harvested from HGPS patients. General hallmarks of aging have been identified as main driving events for the cellular defects of HGPS, including altered nuclear morphology, increased sensitivity to mechanical stress, DNA damage and genomic instability, impairment of chromatin organization, altered gene transcription, dysregulation of signaling pathways and stem cells, and cell death (Lopez‐Otin *et al*, 2013) (Fig 1). However, the specific mechanisms underlying progerin‐induced atherosclerosis have remained elusive because the available models, while showing atherosclerotic lesion formation, do not fully recapitulate the atherosclerotic disease seen in patients (Vidak & Foisner, 2016).

**Figure 1:**
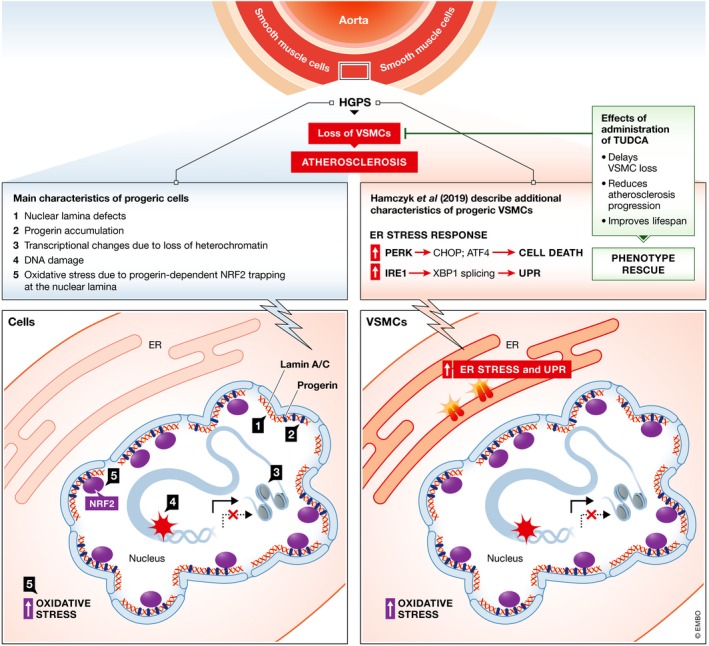
Mechanisms of VSMC loss in HGPS‐dependent atherosclerosis (A) State of the art of cellular and molecular mechanisms taking place in HGPS‐VSMCs from animal models and patients (either primary or iPSC‐derived cells). Nuclear structural defects (1), accumulation of progerin (2), transcriptional changes due to chromatin remodeling (3), and increased DNA damage (4) are the “classical” hallmarks of progeric cells. In addition, activation of the NRF2 pathway and oxidative stress are associated with HGPS. (B) New molecular insights from the work published in this issue, demonstrating that the ER stress response and activation of the UPR play major roles in VSMC death and atherosclerosis in mice with VSMC‐restricted progerin expression. Administration of the chemical chaperone tauroursodeoxycholic acid (TUDCA) is sufficient to rescue cell homeostasis, reduce atherosclerosis progression, and improve lifespan.

On this point, studies conducted on induced pluripotent stem cells (iPSCs) derived from cells harvested from HGPS patients (HGPS‐iPSCs) have revealed more specific mechanisms for the vascular defects in HGPS. For example, progerin‐induced cell death was shown to occur in VSMCs and endothelial cells (ECs) differentiated from HGPS‐iPSCs, via PARP1 downregulation and sustained [Ca2^+^]i elevation driven by transient receptor potential vanilloid 2, respectively (Lo *et al*, 2014; Zhang *et al*, 2014). Studies by Hamczyk *et al* provide an *in vivo* counterpart to these *in vitro* investigations: The authors had previously generated three atherosclerosis‐prone HGPS mouse models—introducing ubiquitous, VSMC‐, or myeloid cell (macrophage)‐restricted expression of progerin into apolipoprotein E‐deficient (ApoE^−/−^) mice—finding that VSMC‐restricted progerin expression was sufficient to accelerate atherosclerosis, probably with activation of cell death as the cause of VSMC loss in the aorta (Hamczyk *et al*, 2018). Their current study furthers the understanding of the underlying mechanisms: Starting from a transcriptional analysis of pre‐disease aortic medial cells, they identify four significantly altered major common pathways: fibrosis, nuclear factor erythroid 2‐like (NRF2)‐mediated oxidative stress, the ER stress response, and the unfolded protein response (UPR).

The NRF2 pathway is a key driver of HGPS‐dependent aging, with repression associated with increased chronic oxidative stress. Indeed, “sequestration” by progerin of NRF2 transcription factor inhibits antioxidant transcription, leading to accumulation of reactive oxygen species and possibly also contributing to activation of the ER stress response (Kubben *et al*, 2016). Interestingly, activation of the UPR may also activate NRF2. The involvement in atherosclerosis of the ER stress response and the UPR is not new. They have been proposed in mechanisms linking the immune response to changes in cell metabolism occurring in the disease. In brief, under conditions that stress the ER, UPR proteins are activated as part of a protective mechanism against ER stress and preserving cell homeostasis.

The UPR signals through three protein branches, mediated by protein kinase RNA‐like ER kinase/eukaryotic translation initiation factor 2‐alpha kinase 3 (PERK/EIF2AK3), inositol‐requiring enzyme‐1 (IRE1), and activating transcription factor‐6 (ATF6). In healthy cells, these are kept inactive by binding to the chaperone BiP. However, when activated, they act cooperatively to reduce protein synthesis, accelerate protein degradation, and promote ER protein folding. When ER stress is not resolved, apoptotic and inflammatory responses are triggered (Hotamisligil, 2010). Alterations of the ER are known to play an important role in macrophages, key immune effectors in vascular lesions; however, this pathway has never been associated before with progerin‐mediated responses in isolated VSMCs. In their study, Hamczyk *et al* (2019) show that upstream regulators of UPR pathways are activated in progerin‐expressing VSMCs. Indeed, they found activation of genes downstream of the main UPR sensors IRE1 and PERK; cell death was the consequence, leading to the VSMC loss observed in the progeric mice.

Considering the role that macrophages have in atherosclerosis, it is surprising that mice with macrophage‐restricted progerin expression did not present with an HGPS phenotype (Hamczyk *et al*, 2018). Zhou *et al* (2005) have shown activation of ER stress/UPR‐related pathways in macrophages recruited to the vascular lesions in ApoE^−/−^ mice, but UPR was mainly triggered by lipid accumulation, at least at later stages. Interestingly, in a very early phase, UPR activation in macrophages appears to be independent of lipid accumulation, suggesting involvement of other pathophysiological factors, such as inflammatory cells or signals from ECs (Zhou *et al*, 2005). The study by Hamczyk *et al* (2019) demonstrates that the activation of this pathway may occur through a cell‐autonomous mechanism in progerin‐expressing VSMCs, without intervention of paracrine factors or activation of the immune system, and that this probably takes place secondarily together with activation of the cell death program. Both progerin accumulation and ER stress are associated with inflammation, two processes strictly dependent upon each other. The immune response is indeed central in atherosclerosis, and it is altered also in HGPS patients and related models. Progeric cells and HGPS mouse models have elevated NF‐kB activation and increased levels of inflammatory markers and cytokines, findings that support a link between the nuclear lamina and inflammation, and thus indicative of a central role of inflammation in accelerated aging (Osorio *et al*, 2012). However, although the activation of an adaptive inflammatory response is likely to occur also in progerin‐expressing VSMCs, data from RNA sequencing shown by Hamczyk *et al* (2019) identify the ER stress response and the UPR as the two most prominent pathways in HGPS‐related atherosclerosis.

Finally, the authors demonstrate that alleviation of ER stress is sufficient to reduce VSMC loss in the aorta: Indeed, administration of the chemical chaperon tauroursodeoxycholic acid (TUDCA) inhibited pro‐apoptotic gene expression, blunted atherosclerosis progression, and prolonged the lifespan of mice with VSMC‐selective progerin expression (Hamczyk *et al*, 2019), which most likely die from atherosclerosis‐related causes (Hamczyk *et al*, 2018). This is a particularly important finding since it confirms the therapeutic value of intervening at the two pathways in VSMCs. Thus, new avenues may be envisaged not only for the treatment of atherosclerosis in HGPS patients but also for physiological aging.
